# A historical perspective of embryo-related technologies in South America

**DOI:** 10.21451/1984-3143-AR2018-0016

**Published:** 2018-08-03

**Authors:** João Henrique Moreira Viana, Ana Cristina Silva Figueiredo, Romany Louise Ribeiro Gonçalves, Luiz Gustavo Bruno Siqueira

**Affiliations:** 1 Embrapa Recursos Genéticos e Biotecnologia, Brasília, DF, Brazil; 2 Universidade Jose do Rosario Vellano, Alfenas, MG, Brazil; 3 Universidade de Brasília, Brasília, DF, Brazil; 4 Embrapa Gado de Leite, Juiz de Fora, MG, Brazil

**Keywords:** cattle, cloning, *in vitro* fertilization, statistics, superovulation.

## Abstract

Livestock production is of great importance for the economy of most South American countries, a region that accounts for 23.0% of the world cattle population (Food Agriculture Organization - FAO, 2017). Not surprisingly, the embryo industry is historically very active in this region, particularly in Argentina and Brazil. The field of bovine embryo transfer underwent a remarkable change in the past two decades in Brazil, mainly due to *in vitro* embryo production (IVEP). Total embryo production increased dramatically, along with constant changes in the main features of the embryo industry - from market niches to mass production, from beef to the dairy sector, from zebu to European breeds. Recently, IVEP has also emerged in other South American countries. This review summarizes and describes factors driving the changes in the Brazilian embryo industry and discusses some of the impacts upon other embryo-related technologies.

## Introduction

The embryo industry has been very active in South America in the past 20 years, with several countries reporting data on embryo production and transfers to the International Embryo Technology Society (IETS), mainly in cattle and horses. Until 2008, however, the methodology used by the data retrieval committee pooled data from each region and presented results as totals. Therefore, before 2009 detailed records are only available from Brazil and Argentina. The IETS reports published afterwards show data for the production of bovine embryos, either *in vivo* or *in vitro*, from the following countries: Argentina, Brazil, Chile, Colombia, Ecuador, Peru, and Uruguay, as well as reports on the export of embryos to Ecuador and Paraguay (Stroud, 2011, 2012; and Perry, 2013, 2014, 2015, 2016). Thus, there are reports of cattle embryo transfer (ET) activity in the past eight years in most of the South American countries. These countries encompass 92.8% of the cattle herd within this region (FAO, 2017), so available data provides a good picture of the ET activity in South America.

Argentina and Brazil have both the largest cattle herds (51,646,544 and 212,366,132 heads in 2014, respectively) and the most active embryo industries in South America. They were consistently ranked among the top countries doing ET in the past 20 years. Brazilian embryo industry has undergone a dramatic increase between 2002 and 2012 (+642.7, or about 55% per year), primarily due to the commercial use of *in vitro* embryo production (IVEP; Viana *et al*., 2017). On the other hand, the use of IVEP in Argentina increased in the past five years and only in 2016, transfers of *in vitro* produced (IVP) embryos reached a 5-digit number (20,234), overtaking *in vivo* derived (IVD) embryos (15,586).

**Figure 1:**
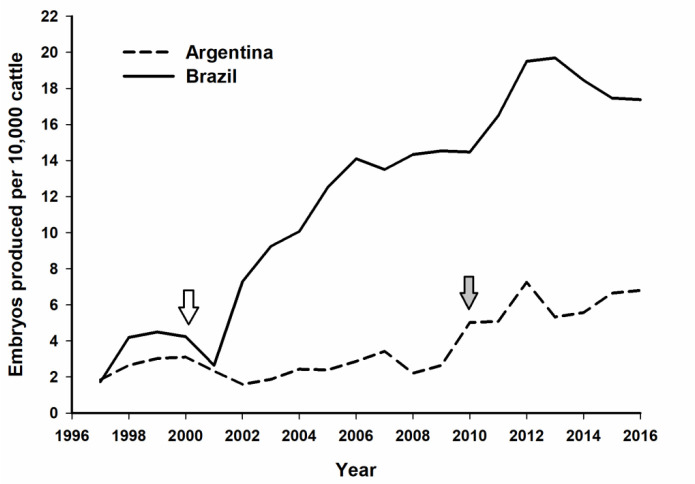
Embryo production relative to cattle population (embryos per 10,000 heads) in Argentina (dashed line) and Brazil (solid line) over the past 20 years. The arrows indicate when the commercial use of IVP embryos was first reported in Brazil (white) and Argentina (grey).

The earlier adoption of *in vitro* embryo technologies was the main reason for the divergence in embryo production trends between the two countries after 2001 (Fig. 1). Taking into account the ratio between embryo production and cattle population in 2014, Brazil ranks 11th in the world, whereas Argentina is the 22nd (Viana *et al.*, 2017). Nevertheless, in both countries the use of IVEP was associated with an increase in total embryo production in cattle.

Argentina and Brazil have also a very active horse embryo industry, reporting an average of 7,400 and 12,840 embryos collected per year, respectively, from 2006 to 2015. Note that there are some missing data for both countries in a couple of the years mentioned, which limits the characterization of the scenario and trends for this species. There are also occasional reports on ET activity in sheep, goats, and alpacas in South America (Perry, 2013, 2014, 2015, 2016). Gathering data from species other than cattle and horse has been a challenge over the years and the numbers reported undoubtedly underestimate the use of embryo technologies in small ruminants in this region.

In summary, bovine is the most important species for the South American embryo industry and Brazil is the main player for the development of embryo technologies in this region, considering both the total numbers of embryos and launching novel trends, such as the earlier use of IVEP in large scale. Thus, many of the aspects of ET development discussed in this paper are supported by data in cattle and from Brazil.

## The effect of IVEP in South America embryo industry

Perhaps the most noticeable features of the embryo industry in South America were the changes introduced by the adoption of *in vitro* technologies. The history of IVEP in Brazil goes back to the early 1990`s, when different research groups started to focus on these technologies. The first calf produced *in vitro* in Brazil was born in 1993, followed by the birth of the first zebu IVP calf, and then the first calf from a cryopreserved IVP embryo (Rubin, 2005). However, there are no official reports about the commercial use of IVEP in Brazil until 1997, and less than 100 IVP embryos were transferred per year in 1998 and 1999.

Meanwhile, IVEP was already intensively used in other continents. By the middle of the 1980’s, several commercial IVEP laboratories were developed in North America and Europe (Faber *et al.*, 2003) and, by 1997, IVP embryos corresponded to 12.6% of all transfers in Europe and 17.5% in Asia (Thibier, 1998). Europe was the region leading the use of IVEP, with 59.9% (18,380 of 30,569) of all IVP embryos transferred worldwide in 1997. In this scenario, although Brazil and Argentina usually ranked within the top five countries in the transfer of IVD embryos outside Europe and North America, there was still no clear sights on the significant shift in the embryo industry scenario that was about to come.

**Figure 2:**
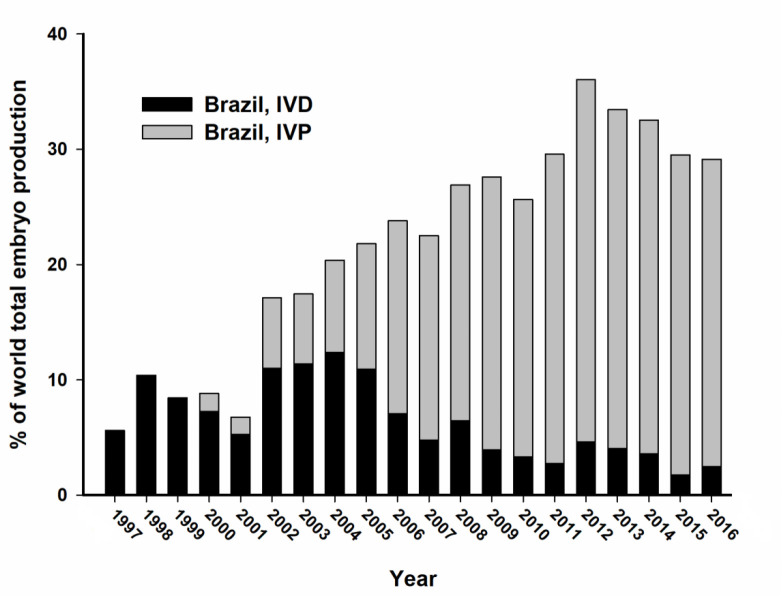
Brazilian contribution to cattle embryo production in the world, in the past 20 years. The columns for each year were subdivided in the percentages corresponding to embryos produced *in vivo* (IVD, black) or *in vitro* (IVP, grey).

**Figure 3:**
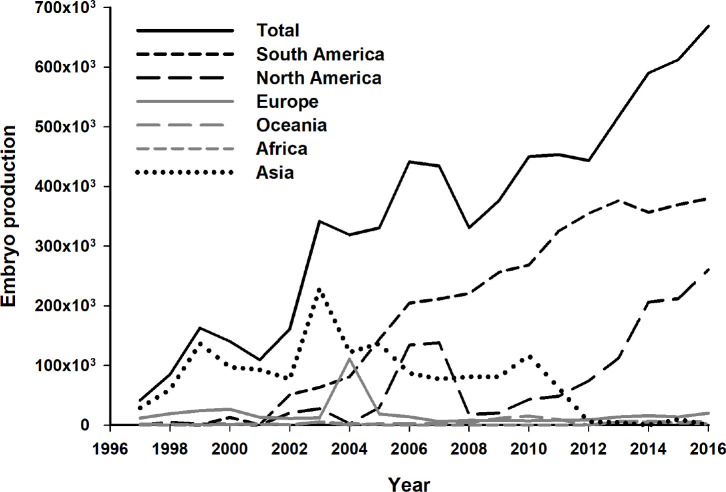
Total bovine embryo production (*in vivo* plus *in vitro*) in the world (solid black line) and divided into different regions (grey, dashed, and dotted lines) ordered according 2016 rank (higher to lower), in the period 1997- 2016. Adapted from Perry (2017).

The whole picture started to change in 2000, when Brazil first reported the production of more than 10,000 IVP embryos. A consistent growth in commercial IVP boosted South American numbers. Only two years later, in 2002, this region already accounted for 58.4% (48,670 of 83,329) of the IVP embryos transferred worldwide (Thibier, 2003). In spite of the usual fluctuations in numbers among different regions through the years, since 2006 Brazil is responsible for >50% of the IVP embryos worldwide. In fact, the adoption of IVEP was responsible for the remarkable participation of Brazil in the world total embryo production after 2002 (Fig. 2). The growth in the use of IVEP in other regions, as currently observed in North America (Fig. 3), will probably increase world total numbers and, consequently, balance the participation of the main players in the world’s embryo industry.

It is important to highlight that IVEP changed not only the magnitude of ET numbers, but also the *modus operandi* of the embryo industry in Brazil and, together with its emergence in other countries, is likely to cause similar effects elsewhere. Since 2005, IVP replaced superovulation as the technique of choice for bovine embryo production (Viana *et al.*, 2012) and the rapid expansion of commercial IVP laboratories pushed traditional ET companies to embrace the new technology. The embryo production process, previously centered in the veterinarian practitioner, now resembles a complex and multi-step production line, which requires technicians with a number of distinct skills.

With the use of transvaginal ultrasound-guided follicle aspiration (OPU), the availability of cumulus- oocyte complexes was no longer a bottleneck because multiple donors can be collected to achieve the number of oocytes predicted to be necessary to generate the required number of embryos. Conversely, the low cryotolerance of IVP embryos have made the availability of suitable recipients a critical factor within ET programs. The increasing efficiency of embryo production *in vitro* (Watanabe *et al.*, 2017), as well as the use of IVEP in large scale (Pontes, 2010, 2011) affected the costs of IVP embryo-related services, the profit margins, and consequently the business model adopted by commercial companies.

## The factors driving the embryo market in Brazil

The reasons behind changes in the Brazilian embryo industry in the past two decades are complex and frequently misunderstood. Embryo technologies have been intensively studied over the past decades and a number of comprehensive review articles have addressed many practical aspects of IVEP. It has some advantages, when compared to embryo production *in vivo* by superovulation, such as the shorter interval between embryo production cycles, the possibility of collecting pre-pubertal or pregnant donors, and of generating contemporaneous calves from the same dam using multiple sires, among other benefits (Galli *et al.*, 2001; Hansen and Block, 2004; Thibier, 2005).

The efficiency of *in vitro* maturation, fertilization, and embryo culture, however, is generally low (Lonergan and Fair, 2008). Moreover, artificial *in vitro* culture conditions can alter epigenetic marks (Urrego *et al.*, 2014) and affect embryonic gene expression (Lonergan *et al.*, 2006). Altogether, these factors affect the quality and cryotolerance of IVP embryos (Lonergan *et al.*, 2006) and have also been associated with higher pregnancy losses, errors in fetal programming (Chen *et al*., 2015; Siqueira *et al*., 2017), and abnormal offspring phenotype, a condition frequently referred to as the large offspring syndrome (Farin *et al.*, 2006). Besides, IVEP requires a much more complex and expensive laboratory infrastructure, with direct impact upon production costs. In this scenario, it was reasonable to consider IVEP as an expensive and uneconomic technology (Gordon, 2003), with a perspective of use as a complement to multiple ovulation and embryo transfer (MOET) programs, particularly for donors that do not respond to superovulation or presenting abnormalities in the reproductive tract (Faber *et al.*, 2003). So, what made IVEP so successful in South America?

Despite the known flaws of the technology, commercial IVEP in Brazil was initially supported by the high demand and high prices of selected sires and dams, especially high-genetic merit oocyte donors of zebu breeds (Viana *et al*., 2012). The focus back then was to produce superior animals for breeding programs, as occurred in other parts of the world (Galli *et al.*, 2003; Smeaton *et al.*, 2003). Nevertheless, particularities of the Brazilian embryo industry at that time generated a *virtuous circle* for IVEP. The high number and quality of cumulus-oocyte complexes (COC) retrieved from zebu breeds (Pontes *et al.*, 2011), for example, resulted in a high embryo yield per session. This led to a reduction in pregnancy costs, which in turn stimulated an increase in the use of IVEP, and the scale effect to promoted further declines in the price of embryos and services. Thus, the technology progressively became economically viable for a greater number of breeds and farmers. Finally, development of IVEP promoted the parallel growth of a chain of suppliers of veterinary services, hormones, IVP media, equipment, disposables, recipients, etc; contributing to a cost reduction and improvements in logistics.

**Figure 4:**
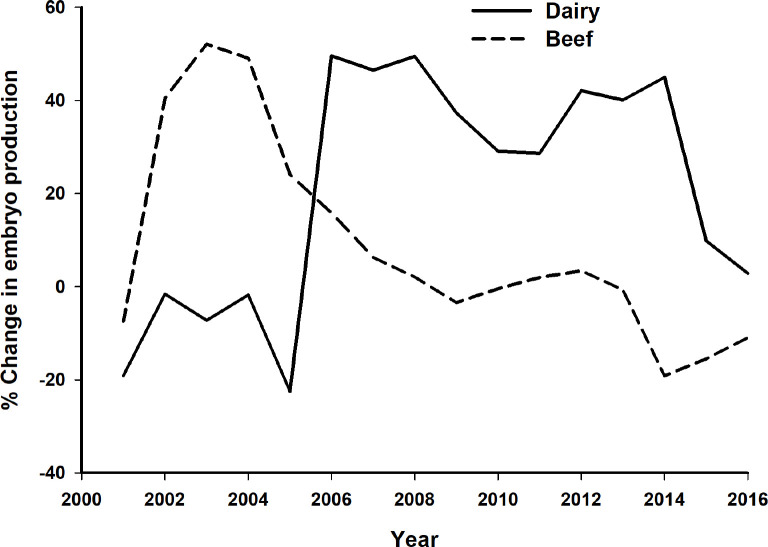
Proportion of changes in the Brazilian embryo production, according to sector (dairy: solid line; beef: dashed line), in the period 2001-2016. Values were calculated based on the variation from the previous year and data was corrected using moving means (3-year average) to reduce the effect of occasional fluctuations.

Three phases of IVEP development in Brazil have been previously described (Viana *et al.*, 2012). The initial period described in the paragraph above was followed by two distinct growth cycles (Fig. 4). First, beef breeds, mainly Nelore, accounted for 82.7% of all embryos transferred in Brazil in 2005 (Viana *et al.*, 2012). This growth cycle was probably associated with a repressed demand for young sires, if one takes into account that in 2008 only approximately 6% of beef cows and heifers were artificially inseminated in Brazil (Baruselli *et al.*, 2012). The second cycle was characterized by the use of IVEP in dairy breeds, particularly in Girolando (Gir x Holstein crossbreds). The availability of commercial X-sorted semen after 2005 was a turning point for the development of IVEP in dairy breeds. Previously, depending on the breed, sire, and culture conditions, the use of conventional semen for IVF resulted consistently in a higher proportion of male births (Alomar *et al.*, 2008; Camargo *et al.*, 2010; Rubessa *et al.*, 2011), which did not meet the expectations of dairy farmers. Therefore, the lack of X-sorted semen in the early 2000’s may explain why the IVEP growth cycle occurred later in dairy, if compared to the early growth in beef breeds.

The emergence of IVEP in dairy breeds highlighted two major changes in the Brazilian embryo industry features. Firstly, IVEP became an alternative for large scale production of replacement heifers, particularly in crossbred herds (Pontes *et al.*, 2010), instead of a reproductive tool restricted to elite animal breeding programs and high-value donors. This represents an important shift in the perception of the potential (and therefore the impact) of IVEP outside Europe and North America - until recently, this technology was referred to as of little application in cattle breeding, particularly in developing countries (Rodriguez-Martinez, 2012). Secondly, the use of *Bos taurus* and crossbreds for IVEP increased dramatically and, in 2013, overcame the production of embryos in pure breed *Bos indicus* (Viana *et al.*, 2017).

More than a simple change in market demand, this shift towards *Bos taurus* suggests that the technical and operational improvements in IVEP made the technique economically interesting even for breeds with lower oocyte yields. Thus, the high number of COC recovered from breeds such as Nelore that might have supported the early emergence of IVEP in Brazil, was no longer a bottleneck for the adoption of *in vitro* technologies. This hypothesis is supported by the late development of IVEP in Argentina (Fig. 1), in which *Bos taurus* breeds are predominant. The increase in embryo production in dairy and *Bos taurus* breeds also turned the South American embryo industry more similar to the North American, with a relative balance between dairy and beef (Table 1).

A parallel development convergent with the new demands of the embryo industry was related to protocols for synchronization of ovulation. These protocols were initially developed for timed artificial insemination (TAI) and currently account for most of AI breedings in Brazil (Baruselli *et al.*, 2012; Sartori *et al.*, 2016). Protocols for timed embryo transfer (TET) were soon adapted for the preparation of embryo recipients (Baruselli *et al*., 2011; Bó *et al*., 2011), and are currently being used in commercial ET programs, with results similar to those obtained using TAI (Pellegrino *et al*., 2016). The main advantage for the use of TET protocols is to increase the synchrony between the embryo developmental stage and the recipient’s uterus, required for achievement of higher pregnancy rates after ET, besides eliminating the well-known problems of estrous detection efficiency (Senger, 1994). Protocols for TET were particularly useful due to the preference for the transfer of fresh IVP embryos (77.9% in South America in 2016; Perry, 2017) and the need to optimize the use of recipients in dairy herds, usually smaller than beef operations. This also have certainly contributed to the long-lasting cycle of growth of IVEP in dairy breeds.

**Table 1:** World embryo production in 2016, according to region, sector (dairy or beef) and technology (IVD, *in vivo* derived; or IVP, *in vitro* produced).

	Dairy			Beef		
Region	IVD	IVP	Total (%)	IVD	IVP	Total (%)
Africa	240	0	240 (3.8)	3,863	2,167	6,030 (96.2)
Asia	13,226	0	13,226 (11.7)	99,372	0	99,372 (88.3)
Europe	99,693	16,678	116,371 (78.2)	29,184	3,296	32,480 (21.8)
NA	111,575	136,204	247,779 (41.8)	220,677	124,370	345,047 (58.2)
Oceania	160	1,956	2,116 (15.3)	7,332	4,364	11,696 (84.7)
South America	17,552	194,357	211,909 (49.6)	29,764	185,445	215,209 (50.4)
Total	242,446	349,195	591,641 (45.5)	390,192	319,642	709,834 (54.5)

## Other embryo-related technologies

The growth of embryo production in Brazil and, particularly, the emergence of IVEP have had direct and indirect effects upon other embryo-related technologies, bringing new challenges for research, but also new market opportunities. One of the indirect consequences was the increased availability of laboratory infrastructure, as well as of qualified technicians. The first Brazilian *in vitro* fertilization company was established in 1998 and, by 2016, the Brazilian Ministry of Agriculture and Livestock have already registered >50 IVEP laboratories (MAPA, unpublished data). This network of laboratories is a platform for the development of other technologies, particularly those that require substantial investments in laboratory equipment (e.g. micromanipulation, intracytoplasmic sperm injection [ICSI], somatic cell nuclear transfer [SCNT], etc.) but have limited commercial use *per se*. In research, IVEP has shifted the focus of some lines of investigation. For instance, due to the replacement of embryo production *in vivo* by *in vitro*, studies on ovarian superstimulation now aim to improve the number and quality of COC per OPU per donor (Vieira *et al.*, 2014; Silva *et al.*, 2017).

A number of research groups are currently developing embryo biotechnologies in Universities and research Institutes throughout South American countries and some of these technologies, such as micromanipulation for embryo sexing or genotyping are beginning to be used commercially. Only recently, however, this kind of data started to be collected by IETS (Perry, 2017) and there are still no comprehensive data for analysis. Bovine clones have been produced in Brazil and in Argentina (Meirelles *et al.*, 2010; Cánepa *et al.*, 2014). Table 2 shows the number of birth records from zebu breeds in Brazil that were derived from SCNT. Despite the low efficiency of embryo reconstruction and very high rates of embryonic loss, abortion, and stillbirths (Chavatte-Palmer *et al.*, 2012), SCNT numbers have increased in the past few years, demonstrating that the demand for the use of the technology has overcome the technical difficulties and, consequently, the high production cost. As expected, most clones were from Nelore (62.5%) and Gir (26.9%), which are the most important beef and dairy zebu breeds in Brazil, respectively. Nonetheless, it is remarkable that the majority of calves (142 of 160, 89.0%) were females and this was true for all breeds. This is probably evidence of a greater interest in cloning high-value oocyte donors, though clones of important sires have also been produced.

**Table 2:** Number of birth records (RGN) from the Brazilian Zebu Cattle Breeders Association that were derived from somatic cell nuclear transfer embryos in the period 2010-2015, stratified by breed, sex, and year of birth.

Breed	2010		2011		2012		2013		2014		2015		Total (%)
Sex	M	F	M	F	M	F	M	F	M	F	M	F	
Gir	0	2	0	0	0	5	2	14	0	6	0	14	43 (26.9)
Guzera	0	0	0	0	0	1	0	2	0	3	0	0	6 (3.8)
Nelore	0	3	1	22	3	11	3	11	3	5	6	32	100 (62.5)
Tabapua	0	0	0	0	0	0	0	6	0	0	0	0	6 (3.8)
Brahman	0	0	0	0	0	2	0	3	0	0	0	0	5 (3.1)
Total	5		23		22		41		17		52		160 (100)

From Associação Brasileira dos Criadores de Zebu - ABCZ, 2017.

## Conclusions

Over the past few decades, embryo industry in South America has been very active, particularly in cattle, with some particular differences among countries. The adoption of IVEP in this region directed significant changes in total embryo production, as well as in the main features of the cattle embryo industry. This phenomenon was first observed in Brazil, which allowed this country to become a reference in the use of *in vitro* embryo technologies and supported the development of other embryo-related technologies. Finally, as IVEP is currently in open expansion in other countries, similar effects are likely to be observed in those regions as well.
